# Exome Sequencing Identifies Compound Heterozygous Mutations in CYP4V2 in a Pedigree with Retinitis Pigmentosa

**DOI:** 10.1371/journal.pone.0033673

**Published:** 2012-05-31

**Authors:** Yun Wang, Liheng Guo, Su-Ping Cai, Meizhi Dai, Qiaona Yang, Wenhan Yu, Naihong Yan, Xiaomin Zhou, Jin Fu, Xinwu Guo, Pengfei Han, Jun Wang, Xuyang Liu

**Affiliations:** 1 Ophthalmic Laboratories & Department of Ophthalmology, State Key Laboratory of Biotherapy, Translational Neuroscience Center, West China Hospital, Sichuan University, Chengdu, Sichuan Province, People’s Republic of China; 2 Shenzhen Eye Hospital, Jinan University, Shenzhen, People’s Republic of China; 3 BGI-Shenzhen, Shenzhen, Guangdong Province, People’s Republic of China; Innsbruck Medical University, Austria

## Abstract

Retinitis pigmentosa (RP) is a heterogeneous group of progressive retinal degenerations characterized by pigmentation and atrophy in the mid-periphery of the retina. Twenty two subjects from a four-generation Chinese family with RP and thin cornea, congenital cataract and high myopia is reported in this study. All family members underwent complete ophthalmologic examinations. Patients of the family presented with bone spicule-shaped pigment deposits in retina, retinal vascular attenuation, retinal and choroidal dystrophy, as well as punctate opacity of the lens, reduced cornea thickness and high myopia. Peripheral venous blood was obtained from all patients and their family members for genetic analysis. After mutation analysis in a few known RP candidate genes, exome sequencing was used to analyze the exomes of 3 patients III2, III4, III6 and the unaffected mother II2. A total of 34,693 variations shared by 3 patients were subjected to several filtering steps against existing variation databases. Identified variations were verified in the rest family members by PCR and Sanger sequencing. Compound heterozygous c.802-8_810del17insGC and c.1091-2A>G mutations of the CYP4V2 gene, known as genetic defects for Bietti crystalline corneoretinal dystrophy, were identified as causative mutations for RP of this family.

## Introduction

Retinitis pigmentosa (RP) is a heterogeneous group of progressive retinal degenerations characterized typically by pigmentation and atrophy in the mid-periphery of the retina. It was estimated to affect 1 in 3500 in the general population [1], [2]. Symptoms for RP include night blindness, tunnel vision and bone-spicule pigmentation in retina.

Considerable clinical and genetic heterogeneity was demonstrated in RP patients, with wide variations in age of onset, severity, clinical phenotype, rate of progression and pattern of inheritance. Genotype-phenotype correlations are not strong enough to predict for RP. About 20–30% of patients with RP also presented with non-ocular disorders such as hearing loss, obesity, and cognitive impairment. Such cases fall within more than 30 different syndromes [3].

Over 50 genes have been identified to cause RP, but still only explain no more than half of the clinical cases [3]. Therefore, there has been limited success with approaches of screening of known candidate genes for RP by conventional Sanger sequencing. Fortunately, exome sequencing technique has come to the aid by enabling the identification of disease-associated mutations by sequencing the whole exome of a small number of affected individuals [4]–[6].

In the present study, disease-associated mutations were identified in a large Chinese family with RP complicated with congenital cataract, corneal thinning and high myopia using the exome sequencing techniques.

## Materials and Methods

### Subjects and Clinical Assessment

Twenty two family members underwent complete ophthalmologic examinations, including slit-lamp biomicroscopy, fundus examination, fundus fluorescein angiography, optical coherence tomography (OCT) for assessment of retinal thickness, B-scan ultrasonagraphy for detection of vitreous and retina, central corneal thickness (CCT) and full-field flash electroretinography (ERG). Written informed consent was obtained in accordance with the Declaration of Helsinki before blood samples were taken for analysis (see attachment for details). The study was approved by West China Hospital, Sichuan University Institute Review Board.

### DNA Extraction

Venous blood samples were obtained from twenty two family members in EDTA Vacutainers. Genomic DNA was extracted from 200 µl peripheral venous blood using Qiamp Blood DNA mini Kit (Qiagen, Hilden, Germany) according to the manufacturer’s instructions. DNA samples were stored at −20°C until used. DNA integrity was evaluated by 1% agarose gel electrophoresis.

### Mutational Screening

Polymerase chain reaction (PCR)-based, direct sequencing was used in the analysis. A number of candidate genes previously shown to be mutated in RP patients including RP1, RP2, RPGR, RHO, RDS, ROM1, TULP1 and RPE65 were sequenced. These genes were known to be frequently involved in autosomal dominant, recessive or X-linked RP [3], [7]. Intronic primers flanking the exons of the candidate genes were designed based on gene sequences of RP1 (GenBank NG_009840.1), RP2 (NG_009107.1), RPGR (NG_009553.1), RHO (NG_009115.1), RDS (NG_009176.1), ROM1 (NG_009845.1), TULP1 (NG_009077.1) and RPE65 (NG_008472.1) synthesized by BGI-Beijing, Beijing, China. DNA fragments were then amplified by PCR using a MyCycler thermocycler (Bio-Rad, Hercules, CA) under the following conditions: 1 µl dNTP (2 mmol/L), 5 µl 10× buffer (containing MgCl_2_, 210 mmol/L), 0.5 µl primer (20 pmol/µl), 3 µl polymerase (5 U/µl) and 5 µl genomic DNA (70 ng/µl). An aliquot of 5 µl of PCR product was subjected to electrophoresis on 1.5% agarose gel to confirm successful DNA amplification.

Purified PCR products were directly sequenced using an ABI 377XL automated DNA sequencer (Applied Biosystems, Foster City, CA). Sequence data were compared pair-wisely with the related Human Genome database.

### Exome Sequencing

The exome sequencing was employed in this study to identify the disease-associated genes based on the following reasons. Firstly, given the fact that the father II1 was deceased 20 years ago and his affected status cannot be ascertained, the exact inheritance pattern cannot be decided with certainty. Secondly, undertaking Sanger sequencing of further RP-associated genes would not be cost-effective. Thirdly, the condition in this family might be due to mutations in a gene not previously reported to be associated with RP.

Exome sequencing was performed on 3 patients (III2, III4, and III6) and II2 (the mother of all the patients) by BGI Inc., Shenzhen, China. The reason for choosing the mother (II2) was that, the data from her was essentially needed in almost all inheritance models including the autosomal recessive model, in which the mother was a carrier. Thirty µg human genomic DNA was extracted from peripheral venous blood samples of each participant. Agilent SureSelect target enrichment system (44 Mb) was used to collect the protein coding regions of human genome DNA. It covered 18134 genes in the Consensus Coding Sequence Region database 2008(http://www.ncbi.nlm.nih.gov/projects/CCDS/). The qualified genomic DNA samples were randomly fragmented on a Covaris Acoustic System, before adapters were ligated to both ends of the resulting fragments. The adapter-ligated templates were purified by Agencourt AMPure SPRI beads. Fragments with insert size about 250 bp were excised. Extracted DNA was amplified by ligation-mediated PCR (LM-PCR), purified, and hybridized to SureSelect Biotinylated RNA Library (BAITS) for enrichment. Hybridized fragments were bound to the strepavidin beads, whereas non-hybridized fragments were washed out after 24 h. Captured LM-PCR products were subjected to Agilent 2100 Bioanalyzer to estimate the magnitude of enrichment. Each captured library was then loaded on HiSeq 2000 platform for sequencing. Each captured library was sequenced independently to ensure each sample had at least 30-fold coverage. Raw image files were processed by Illumina Pipeline v1.7 for base-calling with default parameters and the sequences of each individual were generated as 90 bp paired-end reads. We obtained a mean exome coverage of 46×, which provided sufficient depth to accurately call variants at ∼96% of each targeted exome.

### Variant Analysis

The sequencing reads were aligned to the human reference genome (NCBI Build 36.3) with SOAPaligner (soap2.21) [4]. Based on the SOAP alignment results, the software SOAPsnp v 1.05 [8] was used to assemble the consensus sequence and call genotypes in target regions. Data were provided as lists of sequence variants (SNPs and short indels) relative to the reference genome. Identified variants were filtered against the Single Nucleotide Polymorphism database (dbSNP 129, http://www.ncbi.nlm.nih.gov/projects/SNP/snp_summary.cgi), 1000 genome project (www.1000genomes.org/,1094 individuals from the 20101123 sequence and alignment release of the 1000 genomes project), HapMap 8(http://hapmap.ncbi.nlm.nih.gov/) database and YH database [9] (Table 1 and Table 2).

**Table 1:** Number of candidate variants filtered against several public variation databases.

Feature_SNP	Case (III2)	Case(III4)	Case (III6)	Carrier (II2)
Total_SNPs	49303	59461	53966	58596
Functional_SNPs	12410	14137	13142	14053
Filtered_DBsnp	1561	1792	1577	1768
Filtered_DBsnp_1000gene	932	1076	965	1049
Filtered_DBsnp_1000gene_Hapmap	932	1076	965	1049
Filtered_DBsnp_1000gene_Hapmap_YH	911	1044	940	1017

**Table 2:** Number of candidate Indels filtered against several public variation databases.

Feature_Indel	Case (III2)	Case(III4)	Case (III6)	Carrier (II2)
Total_Indels	3929	4524	4316	4450
Functional_Indels	817	916	885	895
Filtered_DBindel	486	533	521	514
Filtered_DBindel_1000gene	189	211	199	205

We collected reads that were aligned to the designed target regions for SNP identification and subsequent analysis. The consensus sequence and quality of each allele was calculated by SOAPsnp. We filter SOAPsnp results as follows: Base quality is more than 20, depth is between 4 and 200, estimate copy number is equal or less than 2 and the distance between two SNPs must be longer than 4.

### Verification of Variants

Sanger sequencing was used to determine whether any of the remaining variants co-segregated with the disease phenotype in this family. Primers flanking the candidate loci were designed based on genomic sequences of Human Genome (hg18/build36.3) and synthesized by BGI-Beijing, Beijing, China. All shared variants of the three affected individuals after filtering were then confirmed by direct polymerase chain reaction (PCR) and analyzed on an ABI 3730XL Genetic Analyzer. Sequencing data were compared pair-wisely with the Human Genome database.

## Results

### Clinical Assessment and Findings

**Figure 1:**
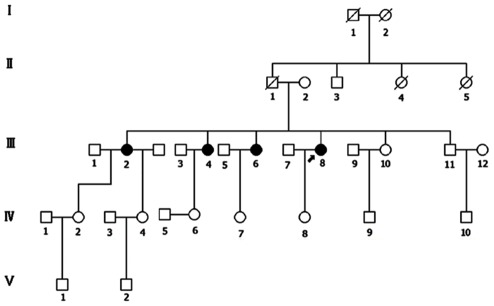
Pedigree of this family with RP. Solid symbols indicate affected individuals. Open symbols indicate unaffected individuals. Arrow indicates the proband and slash indicates deceased person.

A four-generation family from Sichuan Province of China was recruited in this study (Figure 1). Ophthalmic examinations identified 4 affected individuals as RP patients among the 22 examined family members.

**Figure 2:**
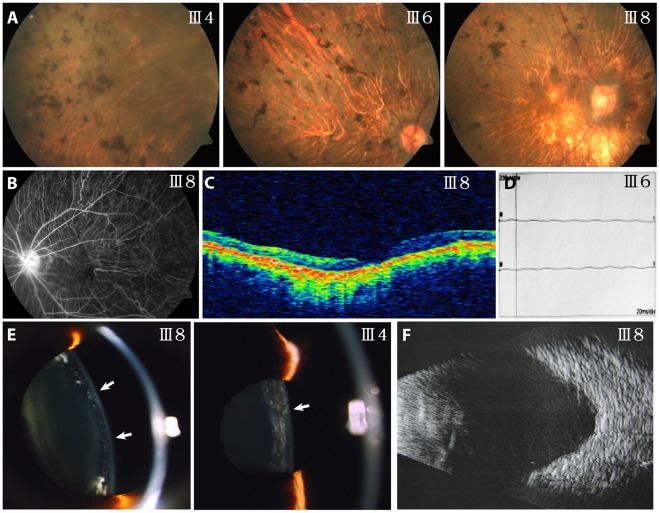
Representative photographs of patients of this family. (A) Fundus photographs showing bone spicule-like pigmentation, optic never head epimembrane and retinal vascular attenuation. Chorioretinal degeneration with peripapillary atrophy was seen. (B) Fundus fluorescein angiography images showing retinal vascular attenuation and chorioretinal atrophy. (C) OCT showing retinal atrophy. (D) ERG records showing no detectable cone and rod responses. (E) Slit-lamp photography showing punctate opacity of the lens as indicted by the arrows. (F) B-scan ophthalmic ultrasonic images showing posterior scleral staphyloma, indicating high myopia.

Affected members of this family exhibited similar clinical features. They suffered from high myopia since about 10 years old. Visual acuity dropped progressively to light perception in their 50 s. Fundus examination and fluorescein angiography in affected patients demonstrated peripheral pigmentation, retinal choroidal atrophy and retinal vascular attenuation in the retina (Figure 2A, 2B). OCT scan demonstrated retinal atrophy (Figure 2C). ERG records showed no detectable cone or rod responses in the patients (Figure 2D). These were consistent with the diagnosis of RP. Punctate opacities of the lens were revealed in affected members under slit-lamp examination (Figure 2E). Corneas of affected members were also found to be thinner. CCT of the unaffected were above 500 µm, while CCT of the patients was in range 460-475 µm on average (Except IV7, who underwent LASIK surgery) (Table 3). B-scan ultrasonagraphy showed posterior scleral staphyloma in all of the patients (Figure 2F), indicating high myopia.

**Table 3:** Phenotype and genotype of subjects.

	Family Member														
	II 2	II 3	III 2	III 4	III 6	III 8	III 10	IV 2	IV 4	IV 5	IV 6	IV 7	IV 8	IV 9	IV 10
Age	84	84	60	57	55	53	50	40	34	35	31	28	30	25	23
Gender	F	M	F	F	F	F	F	F	F	M	F	F	F	M	M
Onset age	–	–	27	25	28	18	–	–	–	–	–	–	–	–	–
Visual Acuity (OD/OS)	N/A	N/A	LP/LP	LP/LP	LP/HM	HM/0.02	0.9/NLP	N/A	N/A	N/A	1.5/1.5	N/A	1.5/1.2	1.0/1.2	0.7/0.3
Corneal Thickness (OD/OS, µm)	N/A	N/A	469/474	468/476	474/476	471/476	507/509	N/A	N/A	525/526	N/A	474/475 (LASIK)	N/A	545/539	525/526
Fundus	Normal	Normal	PP, RVA, RCA	PP, RVA, RCA	PP, RVA, RCA	PP, RVA, RCA	Normal	Normal	Normal	Normal	Normal	Normal	Normal	Normal	Normal
Mutation(s)	c.802-8_810del17insGC	c.1091-2A>G	c.802-8_810del17insGC, c.1091-2A>G	c.802-8_810del17insGC, c.1091-2A>G	c.802-8_810del17insGC, c.1091-2A>G	c.802-8_810del17insGC, c.1091-2A>G	c.802-8_810del17insGC	c.1091-2A>G	c.802-8_810del17insGC	–	–	c.802-8_810del17insGC	c.802-8_810del17insGC	c.802-8_810del17insGC	c.1091-2A>G

Visual acuity shows corrected visual acuity; N/A, not available; PP, peripheral pigmentation; RVA, retinal vascular attenuation; RCA, retinal and choroidal atrophy. The visual acuity in the left eye of III 10 was reduced due to the presence of age related cataract.

### Mutational Screening

Direct sequencing of the *RHO*, *RDS*, *RP1*, *RP2*, *RPGR* (including *ORF15*), *ROM1*, *RPE65* and *TULP1* exons showed no pathogenic mutations in any of the affected individuals in this family. The following SNPs (*rs444772, rs446227, rs414352* of RP1; rs7764439, *rs390659*, *rs425876*, *rs434102* of RDS; *rs5918520* of RPGR) were found in both affected and unaffected members of this family and were shown to have no correlations with the disease.

### Exome Sequencing

Exome sequencing identified 32216 SNPs and 2477 Indels that were shared by the 3 patients. The results were then filtered against several public variation databases, removing all previously reported variants (Table 1, 2). Filtering all exomes for a homozygous mutation causing the disease in the affected sibs (III2, III4, III6), and which was present in heterozygous form in the unaffected mother (II2, “carrier”), Variants satisfying a recessive homozygous inheritance model were not identified. This led us to investigate the possibility of recessive compound heterozygous inheritance. Under the hypothesis of a compound-heterozygous model, we filtered all exomes for variants present in the heterozygous state in all affected individuals for variants and also not present heterozygous in their mother’s exome. It restricted the results to 26 heterozygous variants (Table 4). Heterozygous CYP4V2 c.1091-2A>G was one of the 26 variants, and was known to be responsible for recessive BCD. The mutation was predicted to disrupt the splicing of intron 8, resulting in an in-frame skipping of 45 amino acid–encoding exon 9 [10]–[12].

**Table 4:** Exome sequence variants shared by all affected individuals in homozygous or compound heterozygous states.

Inheritance Model		Homozygous	compound heterozygous	
		Presented heterozygous in carrier (II2)	Presented heterozygous in carrier (II2)	Not presented heterozygous in carrier (II2)
Exome sequence variants sharedby all affected individuals	SNP	0	75	26
	Indel	0	72	22
Disease-associationMutation	–	–	c.802-8_810del17insGC	c.1091-2A>G

As one heterozygous variation was identified from the father side, the other one inherited from the mother (II2) was identified by re-filtered the exome sequencing data for CYP4V2 variations present in all affected individuals and their mother (Table 4). Thirteen variants of the CYP4V2 gene were identified, including two non-synonymous variants c.775C>A and c.802-8_810del17insGC. The former was non-pathogenic [13], whereas the latter harbored a 17 bp deletion including the exon 7 splice-acceptor site, leading to an in-frame deletion of 62 amino acid-encoding exon 7 [13], [14].

**Figure 3:**
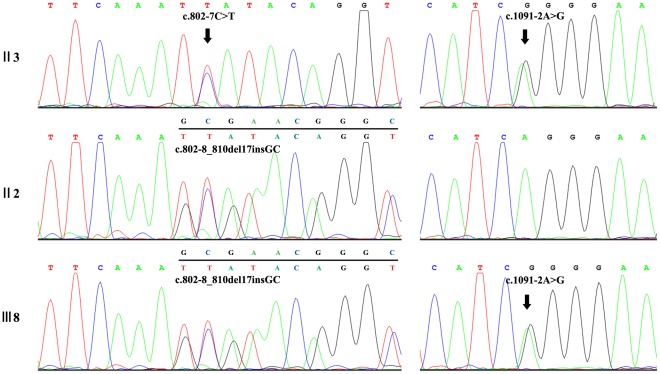
Mutations of the CYP4V2 gene. III8 and other 3 patients harbored compound heterozygous c.802-8_810del17insGC and c.1091-2A>G mutations of the CYP4V2 gene. c.802-8_810del17insGC was carried by the mother II2. c.1091-2A>G mutation was carried by II3, the brother of II1, in one allele, suggesting that the mutation was also carried by II1 and inherited by patients in the fourth generation. Intronic variation c.802-7C>T is non-pathogenic.

All the family members were then screened by PCR amplification and Sanger sequencing for these two mutations, c.802-8_810del17insGC and c.1091-2A>G. Only patients were found to carry both mutations (Figure 3). Phenotypes and underlying mutations of related family members were summarized in Table 3.

## Discussion

In 2004, CYP4V2 defects were identified previously as causative mutations for BCD [11]. The same mutations found in this study have been reported to be associated with an autosomal recessive BCD, which exhibited a totally different phenotype from this pedigree [10]. It is the first time, to the best of our knowledge, to show that mutations in CYP4V2 caused not only BCD, but also RP.

BCD is an autosomal recessive retinal degeneration characterized by multiple tiny glistening crystalline deposits scattered over the fundus. The small glistening crystals can also occur in the corneal limbus and circulating lymphocytes [11], [12], [15]. The molecular basis for BCD remains unclear. Previous studies showed that defects in lipid metabolism were associated with this disease. In BCD patients, the level of polyunsaturated fatty acids (PUFAs) decreased due to the abnormal metabolism of fatty acid precursors, possibly because of the presence of the abnormal lipid-binding protein and enzymes essentially needed in elongation and desaturation of fatty acid [16], [17].

The CYP4V2 gene encodes a member of the cytochrome P450 hemethiolate protein superfamily which is involved in oxidizing various substrates in the metabolic pathway. The CYP4 family is associated with endogenous fatty acid metabolism, with CYP4V2 capabling of hydroxylating the omega-3 PUFAs, including docosahexaenoic acid (DHA) and eicosapentaenoic acid (EPA) [18]. PUFAs are highly enriched in the brain and eye, particularly in the retina [19], playing an important role in regenerating disk membranes of the outer segments of photoreceptor cells [20].

Phenotypically, the patients in this family showed remarkable differences from BCD patients who carried exactly the same mutations [10]. Instead of glistening crystalline deposits, pigment deposits, retinal vascular attenuation and choroidal atrophy were the most significant observations in the fundus. In addition, the patients in our study had younger average age at onset and worse visual acuity than those reported [10]. Interestingly, abnormalities in lipid metabolism was also noticed in RP patients [21]. For example, serum DHA was lower in patients with RP [21]–[24]. DHA deficiency may affect the activity of omega-3 fatty acid desaturation and elongation reactions, and then alter the physical and functional properties of outer segment membranes. Animal studies have shown that reduction of DHA in dietary intake results in abnormal ERGs and visual loss [25], [26]. Clinical trial in RP patients showed that progression of RP could be prevented or slowed down when the patients were treated with DHA [27]. Dietary supplementation of DHA in such patients would by-pass some biosynthetic and transport steps and may restore blood levels of DHA back to normal [28]. All these suggest a link between DHA deficiency and risk of RP, and between CYP4V2 defects and the pathogenesis of RP.

Since the inheritance pattern of this pedigree was not clearly clarified, making the genetic analysis of this pedigree difficult. We presumed autosomal recessive as the most likely inheritance model. Mutational screening for several genes associated with autosomal recessive inheritance failed to identify the causative gene(s). Given the fact that many mutations in at least 50 genes are known to cause autosomal recessive RP (RetNet: http://www.sph.uth.tmc.edu/Retnet/sum-dis.htm) and more to be identified, exome sequencing was employed for genetic analysis of this pedigree. Our results showed that this approach can be used to effectively narrow down candidate genes and to identify genetic defects responsible for Mendelian-inheritance diseases in pedigrees.

The mother II2 in this study was not a real negative control for exome sequencing since she was supposed to be a carrier in the autosomal recessive model. Initial analysis of exome sequencing showed that c.1091-2A>G in CYP4V2 was first carried by the 3 patients (III2, III4, III6); further sequence verification showed that this variation was present not only in another patient (III8), but also in unaffected individuals, including II1’s brother (II3). It was thus presumed that this heterozygous variation was inherited from father II1, and carrying this variation only was not pathogenic. In a compound-heterozygous model, as one heterozygous variation was identified from the father side (II3), the other one inherited from mother (II2) was identified by re-filtered the exome sequencing data for variations present in all affected individuals and their mother. The mutation c.802-8_810del17insGC in CYP4V2 was then identified, since only the four patients carried both c.1091-2A>G and c.802-8_810del17insGC in CYP4V2.

Among the mutations identified in this pedigree, c.1091-2A>G of CYP4V2 was predicted to disrupt the splicing of intron 8, resulting in an in-frame skipping of 45-amino-acid encoding exon 9. [11], [12] The other 3′ splicing acceptor site mutation, c.802-8_810del17insGC was reported as a frequent founder mutation in East Asian populations [13], [29]. The change in this splicing acceptor site was expected to cause an in-frame deletion of 62 amino acid-encoding exon 7, which was confirmed by reverse transcriptase (RT)-PCR [13], [14].

In summary, a RP- associated gene, CYP4V2, was identified by exome sequencing. The phenotype–genotype correlations with regard to CYP4V2 sequence alterations were discussed. Our study highlights the clinical heterogeneity of RP and demonstrates that exome sequencing can be a valuable method to the diagnosis of genetic diseases. Most interestingly, the same compound heterozygous mutations were identified to cause two retinal disorders with totally different phenotypes. The underlying mechanisms need to be further elucidated.
